# Formulation and evaluation of microsphere based oro dispersible tablets of itopride hcl

**DOI:** 10.1186/2008-2231-20-24

**Published:** 2012-09-03

**Authors:** Sanjay Shah, Sarika Madan, SS Agrawal

**Affiliations:** 1 Delhi Institute of Pharmaceutical Sciences & Research (Formerly College of Pharmacy), University of Delhi, Pushp Vihar, Sector III, New Delhi, 110017, India

**Keywords:** Taste masking, Orodispersible tablets, Itopride HCl, Eudragit EPO, Superdisintegrants

## Abstract

**Background:**

The purpose of the present work is to mask the intensely bitter taste of Itopride HCl and to formulate an Oro dispersible tablet (ODT) of the taste-masked drug by incorporation of microspheres in the tablets for use in specific populations viz*.* pediatrics, geriatrics and patients experiencing difficulty in swallowing.

**Methods:**

With this objective in mind, microspheres loaded with Itopride HCl were prepared by solvent evaporation method using acetone as solvent for pH-sensitive polymer, Eudragit EPO and light liquid paraffin as the encapsulating medium. The prepared microspheres were characterized with regard to yield, drug content, flow properties, particle size and size distribution, surface features, in vitro drug release and taste. The ODTs so prepared from these microspheres were evaluated for hardness, thickness, weight variation, friability, disintegration time, drug content, wetting time, water absorption ratio, moisture uptake, in vitro dispersion, in vitro disintegration, in vitro drug release and stability.

**Results:**

The average size of microspheres was found to be satisfactory in terms of the size and size distribution. Microspheres prepared were of a regular spherical shape. Comparison of the dissolution profiles of microspheres in different pH media showed that microspheres having drug: polymer ratio of 1:2 produced a retarding effect in simulated salivary fluid (pH 6.8) and were further used for formulation into ODTs after addition of suitable amounts of excipients such as superdisintegrant, diluent, sweetener and flavor of directly compressible grade.

**Conclusions:**

Effective taste-masking was achieved for Itopride HCl by way of preparation of microspheres and ODTs of acceptable characteristics.

## Introduction

Among the different routes of administration, oral route of administration continues to be the most preferred route due to various advantages including ease of ingestion, avoidance of pain, versatility and most importantly patient compliance. The different dosage forms include tablets, capsules and oral liquid preparations. The important drawback of tablet and capsule dosage forms for pediatric and geriatric patients has been difficulty in swallowing [1]. To overcome this problem, formulators have considerably dedicated their effort to develop a novel type of tablet dosage form for oral administration called “Oro Dispersible Tablet”, (ODT) i.e., one, which disintegrates and dissolves rapidly in saliva without the need of water [2]. United States Food and Drug Administration (FDA) defined ODT as “A solid dosage form containing medicinal substance or active ingredient which disintegrates rapidly usually within a matter of seconds when placed upon the tongue [3].” The disintegration time for ODTs generally ranges from several seconds to about a min.

The two key parameters that need to be considered in the development of ODTs are taste masking of bitter drug and the disintegration time. Various taste-masking technologies have been extensively reviewed [4-6]. Solvent evaporation is a relatively simple and convenient method for the preparation of taste-masked microspheres. The drug particles are surrounded by a polymer which prevent leaching of the drug into the saliva but allow the release of the drug in the stomach. The most widely used solvent systems in solvent evaporation process are methylene chloride/water [7] and acetone/light liquid paraffin [8-11]. This technology has been applied to mask the bitter taste of therapeutic agents such as bacampicilin [8,12] pseudoephedrine [7] and cefuroxime axetil [10].

Itopride Hydrochloride is used for the management of Nonulcer dyspepsia (NUD), gastro-esophageal reflux disease (GERD), gastritis, diabetes gastro paresis and functional dyspepsia. It is a newer gastroprokinetic agent with anti cholinesterase activity as well as D_2_ receptor antagonistic activity and is being used for the symptomatic treatment of various gastrointestinal motility disorders [13]. Itopride HCl is available in market in the form of immediate release tablets/capsules, for e.g. “GANATON®” sold by Abbott Laboratories (Abbott Park, Illinois). Itopride HCl is a highly water soluble and intensely bitter drug. Thus, it was envisaged to mask the taste of Itopride HCl by way of making microspheres prior to formulating ODTs with good mouth feel. To the best of our knowledge, it is the first time such an attempt has been made that would explore the rational approach of incorporating taste-masked microspheres of Itopride HCl in an orally disintegrating tablet.

## Materials and methods

### Materials

Itopride HCl was obtained as a gift from Ranbaxy Laboratories Ltd (Gurgaon, India). Aminoalkyl methacrylate copolymer (Eudragit EPO) was obtained as a gift from Degussa India Private Ltd (Mumbai, India). Magnesium stearate was kindly supplied by Unichem (New Delhi, India). Other excipients that were purchased included mannitol (Pearlitol, CDH (P) Ltd., New Delhi, India), sodium stearyl fumarate (ITM Chem. Pvt. Ltd. Mumbai), Nutra Sweet (Aspartame, Kawarlal and sons, Chennai, India) and crospovidone (Polyplasdone XL-10, Nanz Med Sciences Pharma Pvt. Ltd., Delhi, India). All other chemicals used in the study were of analytical grade and used as received.

### Methods

#### Preparation of microspheres

Microspheres were prepared by the solvent evaporation method previously reported and modified for our purpose [10,12]. Firstly, Eudragit EPO was dissolved in acetone on a magnetic stirrer to obtain uniform mixing. Itopride HCl was then added to the above solution. To this mixture, magnesium stearate was added. The polymer drug solution so obtained was injected into light liquid paraffin at a low stirring speed (200–600 rpm) of mechanical stirrer for about 3 h until all the acetone evaporated. n-Hexane/petroleum ether was added to the system for hardening of the microspheres and to accelerate settling. Microspheres were separated by decantation following filtration through a Whatman filter paper (No. 41). Microspheres were then washed with *n*-hexane and the washed microspheres were dried in an oven maintained at 37°C for 24 h. Dried microspheres were stored at room temperature. Various drug: polymer ratios were selected for the formulation of microspheres (Table 1). The formulation parameters and process parameters for different batches of microspheres were evaluated (Table 2).

**Table 1:** Different ratios of Drug: polymer for preparation of microspheres (mean ± SD, n = 3)

**Batch**	**Microspheres ingredients and process parameters**						Microspheres evaluation parameters *				
	**Itopride HCl (mg)**	**Eudragit EPO (mg)**	**Magnesium stearate (mg)**	**Liquid paraffin (ml)**	**Acetone (ml)**	**Speed (RPM)**	**Yield (%)**	**Average Particle Diameter (μm)**	**Drug content (%)**	**Loading efficiency (%)**	**% release of drug in simulated saliva in 60 sec**
A1	200	200	200	40	2.0	600	69.3 ± 1.2	186.3 ± 2.5	37.86 ± 0.5	94.65 ± 1.2	6.31 ± 0.09
A2	200	400	200	40	2.0	600	72.3 ± 1.5	197.2 ± 2.8	32.02 ± 0.2	96.09 ± 1.3	1.61 ± 0.06
A3	200	600	200	40	2.0	600	77.6 ± 2.1	202.2 ± 3.7	24.32 ± 0.3	97.28 ± 1.6	1.57 ± 0.04
A4	200	800	200	40	2.0	600	75.9 ± 1.7	278.6 ± 4.2	19.23 ± 0.1	96.15 ± 1.4	1.51 ± 0.05
A5	200	1000	200	40	2.0	600	68.3 ± 2.4	327.9 ± 3.6	16.17 ± 0.2	97.05 ± 1.3	1.43 ± 0.07

**Table 2:** Taste-masked Itopride HCl microsphere ingredients, process parameters and evaluation (Mean ± SD, n = 3)

**Batch**	**Microspheres ingredients and process parameters**						**Microspheres evaluation parameters**			
	**Itopride HCl (mg)**	**Eudragit EPO (mg)**	**Acetone (ml)**	**Magnesium sterate(mg)**	**Liquid paraffin (ml)**	**Speed (RPM)**	**(%) Yield**	**Average Particle Diameter(μm)**	**(%) Drug content**	**% Loading efficiency**
B1	200	400	1.6	200	40	600	70.8 ± 1.3	285.5 ± 1.2	24.76 ± 0.4	99.04 ± 0.5
B2	200	400	1.8	200	40	600	81.89 ± 1.4	216.9 ± 1.7	24.05 ± 0.7	96.20 ± 0.07
B3	200	400	2.0	200	40	600	78.19 ± 1.5	197.8 ± 2.4	23.84 ± 0.3	95.36 ± 0.4
B4	200	400	2.2	200	40	600	72.6 ± 1.6	121.1 ± 1.9	23.96 ± 0.5	95.84 ± 0.2
C1	200	400	1.8	80	40	600	Batch Failed			
C2	200	400	1.8	120	40	600	61.59 ± 2.3	283.0 ± 2.6	27.56 ± 0.1	99.21 ± 0.1
C3	200	400	1.8	160	40	600	82.32 ± 2.5	238.5 ± 1.5	25.94 ± 0.2	98.58 ± 0.2
C4	200	400	1.8	180	40	600	80.18 ± 2.1	201.1 ± 3.5	24.83 ± 0.4	96.84 ± 0.4
D1	200	400	1.8	160	20	600	Batch Failed			
D2	200	400	1.8	160	30	600	73.84 ± 1.6	302.5 ± 2.4	26.17 ± 0.6	99.46 ± 0.5
D3	200	400	1.8	160	40	600	82.13 ± 1.7	264.7 ± 1.0	25.96 ± 0.7	98.66 ± 0.7
D4	200	400	1.8	160	50	600	75.58 ± 2.5	182.4 ± 3.1	25.47 ± 0.8	96.80 ± 0.3
E1	200	400	1.8	160	40	200	Batch Failed			
E2	200	400	1.8	160	40	400	84.31 ± 3.0	296.58 ± 2.2	25.36 ± 0 .7	96.35 ± 0.5
E3	200	400	1.8	160	40	600	82.11 ± 2.8	214.60 ± 3.2	25.43 ± 0.09	96.66 ± 0.3
E4	200	400	1.8	160	40	800	77.74 ± 3.2	174.64 ± 2.0	25.44 ± 0.7	96.66 ± 0.2
E5	200	400	1.8	160	40	1000	62.76 ± 2.8	128.5 ± 1.8	24.96 ± 0.4	94.48 ± 0.1

### Characterization of Microspheres

#### Percentage Yield

The prepared microspheres were completely dried in an oven maintained at 37°C for 24 h and then weighed. The percentage yield of microspheres was calculated according to the following equation:

(1) $$\%\;yield=\frac{\mathrm{Pr}acticalyield}{Theoreticalyield}*100$$

#### Drug Loading and Drug content [14]

The microspheres (100 mg) were crushed in pestle-mortar and then stirred with 100 ml 0.1 N HCl (pH 1.2) for 2 hr to ensure complete elution of drug. The readings were taken in triplicate. The Itopride HCl content of the microspheres was calculated using a standard calibration curve prepared with UV-Visible spectrophotometer at 258 nm after suitable dilution.

(2) $$\begin{array}{l} Drug\;content\left(\%\right)=\frac{Weight\;of\;drug\;in\;microspheres}{Weight\;of\;microspheres}\\ \;*100 \end{array}$$

(3) $$\begin{array}{l} Drug\;loading\left(\%\right)=\frac{Weight\;of\;drug\;in\;microspheres}{Weight\;of\;drug\;initially\;added}\\ \;*100 \end{array}$$

### Evaluation of flow properties of microspheres

The prepared microspheres were evaluated for flow properties [15] including bulk density (*D*_*b*_), tapped density *(ρ*_*t*_*)*, Carr’s compressibility index *(I)*[16]*,* Hausner ratio *(H)*[17] and Angle of Repose *(θ).*

#### Micromeritics of microspheres

The size distribution and average size of the microspheres were determined by sieve analysis using American society for testing of materials (ASTM) sieves. A set of 12 sieves ranging in size from 1.18 mm (# 16) to 75 microns (# 200) mounted on a sieve shaker unit was used. Amount of microspheres remaining on each sieve was then weighed and calculated [18].

#### Scanning Electron Microscopy Analysis of the Microspheres

The drug and microspheres were characterized further using a scanning electron microscope (JEOL JSM5200, Japan Electron Optics Ltd., Japan) after gold sputtering. Shapes and surface characteristics of the microspheres were investigated and photographed.

#### Thermal analysis of the microspheres

Differential scanning calorimetric (DSC) experiments were performed on Itopride Hydrochloride, Eudragit EPO and drug-loaded microspheres (Shimadzu TA 60WS, Japan). Accurately weighted samples (2–5 mg) were sealed in flat bottom aluminum pans and heated from ambient to 200°C at a rate of 10°C/min in a nitrogen atmosphere (flow rate, 10 ml/min).

#### Fourier-Transform IR (FTIR) studies

Fourier Transform Infrared (FTIR) scans of Itopride HCl, Eudragit EPO, Physical ad-mixture of drug plus Eudragit EPO and drug-loaded microspheres were recorded (Jasco FTIR-410, Japan). All the discs were prepared in KBr press.

### In vitro *release studies*

In vitro drug release studies were carried out using USP XXIV dissolution apparatus II. [19]Accurately weighed Itopride HCl microspheres (equivalent to 10 mg of Itopride HCl) were added to the medium under test. The test was carried out in pH 1.2 (900 ml) and pH 6.8 phosphate buffer (900 ml) equilibrated at 37 ± 0.5°C. The paddle was rotated at 50 rpm. At specific times of 5, 10, 15 and 30 min, aliquots of the dissolution medium were withdrawn and which replaced with 10 ml of fresh dissolution medium. The collected samples were analyzed using UV spectrophotometer at 258 nm.

#### Preparation of the ODTs

Orodispersible tablets of Itopride HCl microspheres were prepared by direct compression technique using crospovidone as a superdisintegrant. The optimization of tablet disintegration is commonly done by means of the disintegration critical concentration. Below this concentration the tablet disintegration time is inversely proportional to the disintegrant concentration. Above the critical concentration, the disintegration time remains approximately constant or even increased [20]. The selected batch of microspheres were incorporated into tablets on the basis of drug loading so as to give the required dose of 50 mg/tablet. The microspheres were blended along with the other excipients (super-disintegrant, sweetener, flavor and lubricant) and processed to allow direct compression tabletting in a single punch tablet machine (Cadmach, India) using 11-mm round, convex-faced, beveled edge tooling (Panacea tools Ltd., New Delhi).

### Evaluation and characterization of Tablets

Thickness of tablets was assessed using a Vernier caliper. The hardness of the tablets was determined by using Monsanto hardness tester (Pharma Chem. Machineries, India) and about 4–6 kg/cm^2^ was considered adequate for mechanical stability of ODT [21]. Uniformity of weight was also determined as per Indian Pharmacopoeia (IP) [22]. As per USP 30-NF 25, friability of twenty six tablets was determined using Roche Friabilator (EI Products, India) [21]. The India Pharmacopoeia prescribes a friability <1% for good mechanical resistance.

#### Determination of drug content

Six tablets were crushed to a powder and powder equivalent to 10 mg Itopride HCl was taken and dissolved in 0.1 N HCl to extract the active ingredient. The solution was filtered through Whatmann filter paper (no. 41). After suitable dilution with 0.1 N HCl, the drug content was analyzed by UV spectrophotometer at 258 nm [23].

### In vitro *evaluation of tablets*

Wetting time and water absorption ratio of tablets were determined as per method prescribed in the literature earlier [24]. Moisture uptake test was performed in conditions prescribed in literature [25]. In vitro dispersion time was measured by dropping a tablet without disc in a measuring cylinder containing 10 ml of phosphate buffer pH 6.8 (simulated saliva fluid and time required for complete dispersion of a tablet was measured. In vitro disintegration test was carried out using USP XXIV tablet disintegration test apparatus (Pharma Test, Germany) [26]. The time for disintegration of ODTs is generally less than one min and actual disintegration time that patient can experience ranges from 5–30 seconds. In-vitro Dissolution studies [27] were performed using USP XXIV Type II dissolution paddle apparatus (Lab India, DS 8000, India). The dissolution test was performed using 900 ml of 0.1 N HCl buffer at 37 ± 0.5°C. The speed of rotation of paddle was set at 50 rpm. Samples of commercial product GANATON and oral disintegrating tablet (F2) of Itopride HCL (equivalent to 50 mg of Itopride HCL) were introduced in the dissolution medium. The dissolution tests were carried out for 2 h with sampling time intervals of 2, 5, 10, 15, 30, 45, 60, 90 and 120 min respectively. The samples were analyzed using a double beam UV-spectrophotometer and the absorbance was recorded at 258 nm. The in-vitro dissolution studies were performed in triplicate.

### In Vivo *studies*

#### Pharmacokinetic studies

ODT tablet was subjected to bioequivalence studies using albino rabbits and observed plasma concentration was plotted against the time and compared with marketed formulation GANATON.

The experiments were conducted as per CPCSEA (Committee for Prevention, Control and Supervision of Experimental Animals) guidelines. Rabbits (2.0-2.5 kg) of either sex were kept in normal housing conditions and were fed with commercially available diet, sprouted grams and cabbage. The rabbits were used in the study in accordance with a protocol approved by the Institutional ethical committee at DIPSAR protocol number: DIPSAR/IAEC/21/2010.

The rabbits were randomly divided into two groups of three rabbits each. All the rabbits were fasted for 12 h with ad libitum access to water. One group received Marketed product GANATON whereas the other group received the test formulation ODT F2*.* The test tablets were administered in the mouth of rabbit through intra-gastric tube and immediately 5 ml of water was administered to facilitate swallowing of the powder and to prevent it from sticking to the rabbit’s throat while the marketed tablet was crushed prior to administration. Blood sample (0.6 ml) was withdrawn from marginal ear vein into eppendorf tubes containing EDTA at time intervals of 5, 15, 30, 45, 60, 120 min and 4, 6, 8, 12, 16 and 24 h post administration. The blood was immediately centrifuged at 3500 rpm for 30 min at 0°C and plasma was stored at −20°C until HPTLC analysis. To 0.5 ml aliquot of plasma, 0.5 ml of ethyl acetate was added, centrifuged at 3500 rpm for 40 min at 0°C. The supernatant was separated. From this supernatant ethyl acetate was allowed to evaporate. When the samples were dried completely they were reconstituted with mobile phase (chloroform: methanol, 9:1) and kept frozen until analyzed. The plasma drug content was analyzed by HPTLC (CAMAG, Switzerland). Reconstituted sample of 1, 2, 3, 4, 5, 6, 7 and 8 μL was spotted in the bands of 4 mm width using a CAMAG microlitre syringe - volume of 100 μL, on to the precoated silica gel aluminium plate using Linomat V sample applicator. The plate was allowed to develop in linear ascending mode in CAMAG twin trough glass chamber saturated with the mobile phase. The saturation time for mobile phase was chosen at 30mins. The chromatogram run was 8 cm. The developed TLC plate was scanned on a CAMAG TLC Scanner III in the absorbance mode of 258 nm by using a deuterium lamp as a source of radiation. Pharmacokinetic parameters for Itopride following administration were determined from plasma concentration-time data. The pharmacokinetic profile of the formulation was compared with the marketed formulation administered orally to the other group of animals.

The pharmacokinetic parameters, namely, maximum plasma concentration (C_max_) and time to reach C_max_ (T_max_) were obtained directly from the plasma concentration–time data. The area under the plasma concentration– time curve from 0 to 24 h (AUC_0—24 h_) was calculated by the trapezoidal rule.

#### Stability studies

The tablets of the formulation (F2) were subjected to stability studies. During the study period of 40°C/75% RH for a specific time period up to 30 days, several parameters like hardness, friability, in vitro dispersion and drug content uniformity were evaluated at 1, 2, 3 and 4 weeks time interval for possible instability problems [28].

## Results and discussion

The taste masking of Itopride HCl was carried out by coating the drug with Eudragit EPO polymer using solvent evaporation method that is not only a one step process but can be easily controlled and scaled up. Eudragit EPO was used as a taste masking agent because it dissolves at a pH of less than 5 such as in the stomach (pH 1–3) to release the drug [29]. As the polymer does not dissolve in the buccal cavity (pH 5.8-7.4), the coated drug remains intact to produce good taste masking. Magnesium stearate was used to prevent electrification and flocculation in the preparation of microspheres [9,12].

Microspheres were prepared in drug: polymer ratios 1:1, 1:2, 1:3, 1:4, 1:5 as shown in Table 1. The amount of the drug released from the microspheres in simulated salivary fluid in 60 sec was considered. When the drug/polymer ratio was increased from 1:2 to 1:3 or 1:4 or 1:5, microspheres showed almost the same drug release profile in simulated salivary fluid (1.61%, 1.57%, 1.51% and 1.43%, respectively). However, if the ratio was changed from 1:2 to 1:1, a sharp increase of drug release was observed (6.31%). Therefore, the ratio 1:2 was considered to be the most suitable with respect to taste masking, and microspheres consisting of Itopride hydrochloride and Eudragit® EPO (1: 2) were further evaluated for other parameters.

The prime parameters for selection of batches were based on particle size, loading efficiency and drug content and these were evaluated (Table 2). From the average diameter (D_av_) of microspheres determined by sieve analysis, it was found that on increasing the stirring rate, amount of acetone and liquid parafin, the mean diameter of the microspheres was found to decrease. A reduction in stirring speed from 600 to 200 rpm resulted in failure of batch as seen in batch E1. All microspheres varied in the size from batch to batch. Microspheres were spherical in the size range of 300–425 μm (fraction # 40/50) and 212–300 μm (# 50/70, major fraction) and microspheres of other sieve fractions 125–150 μm (# 100/120) and 125–106 μm (# 120/140) were not perfect spheres.

Further, batches of microspheres were evaluated for bulk density, tapped density, angle of repose, Carr’s consolidation index and Hausner ratio. The results of powder flow properties (Table 3) clearly indicated good flow characteristics for batch C3 and E3. Based upon % yield, drug content and loading efficiency, average size, flow properties and estimation of bitter taste of microspheres, batch E3 was considered to be optimum and was therefore selected for further characterization and tabletting.

**Table 3:** Physical properties of Microspheres of Itopride HCl* (Mean ± SD, n = 3)

**Formulation code**	**Evaluation properties**					
	**Angle of Repose (θ)**	**Bulk Density (g/cm**^**3**^**)**	**Tapped density (g/cm**^**3**^**)**	**Carr’s Compressibility index (%)**	**Hausner’s Ratio**	**Flowability**
B2	29.35 ± 1.67	0.58 ± 0.02	0.71 ± 0.12	16.32 ± 0.55	1.56 ± 0.02	Fair
C3	27.21 ± 1.34	0.56 ± 0.15	0.67 ± 0.03	18.49 ± 0.19	1.36 ± 0.01	Good
D3	30.56 ± 1.56	0.61 ± 0.05	0.68 ± 0.04	19.57 ± 0.22	1.28 ± 0.04	Fair
E3	24.87 ± 0.89	0.52 ±0.12	0.65 ± 0.02	14.09 ± 0.44	1.15 ± 0.02	Excellent

**Figure 1:**
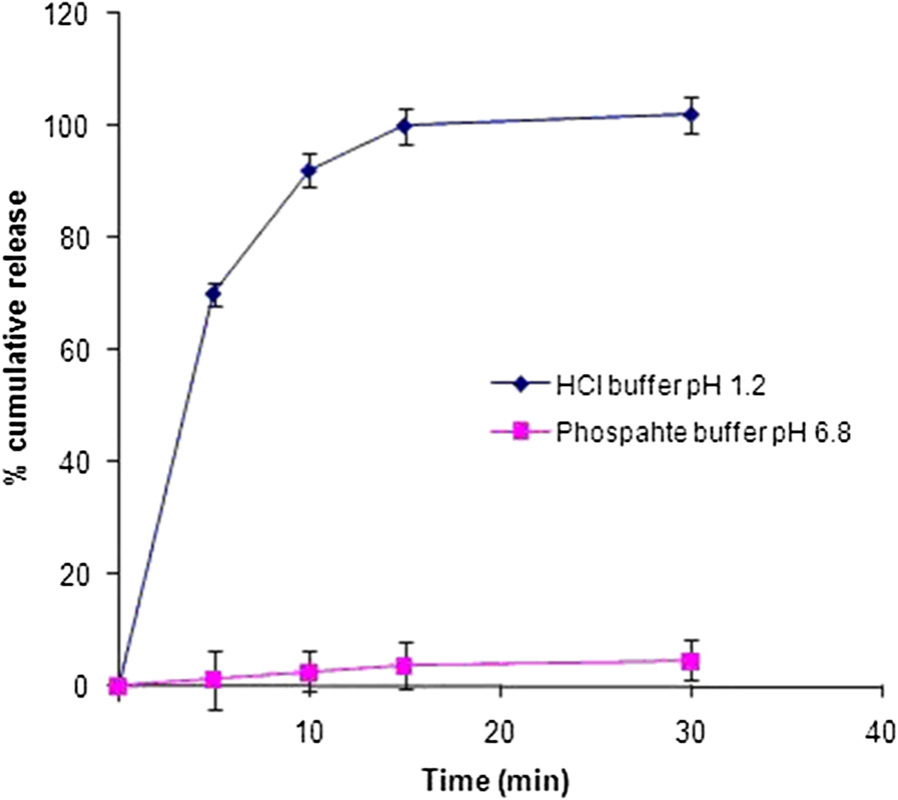
**Dissolution profiles of Itopride HCl taste-masked microspheres in pH 1.2 hydrochloric acid buffer and pH 6.8 phosphate buffer.** Each value represents an average of three determinations. (Mean ± SD,n = 3).

Dissolution studies of Itopride HCl microspheres (Batch E3) were performed in two different pH media (pH 1.2 hydrochloric acid buffer and pH 6.8 phosphate buffer (Figure 1). Eudragit EPO is soluble in an acidic environment by formation of salts. Therefore, drug released from the microspheres very rapidly in pH 1.2 medium such that approximately 90% of the drug was released within 10 min. Drug release in pH 6.8 buffer was comparatively slower than that in pH 1.2 medium. About 2.6% drug released in 10 min in pH 6.8 medium. Eudragit EPO is insoluble at pH greater than 5 but it becomes permeable and allows the release of Itopride HCl. The gastric emptying time ranges from 32–87 min in fasted states for particles with a size in the micron range and the corresponding value is 34–75 min in fed state [30]. Therefore, it is expected that as soon as the polymer dissolves in the acidic contents of stomach, drug will be released in stomach followed by absorption from the gastrointestinal tract.

**Figure 2:**
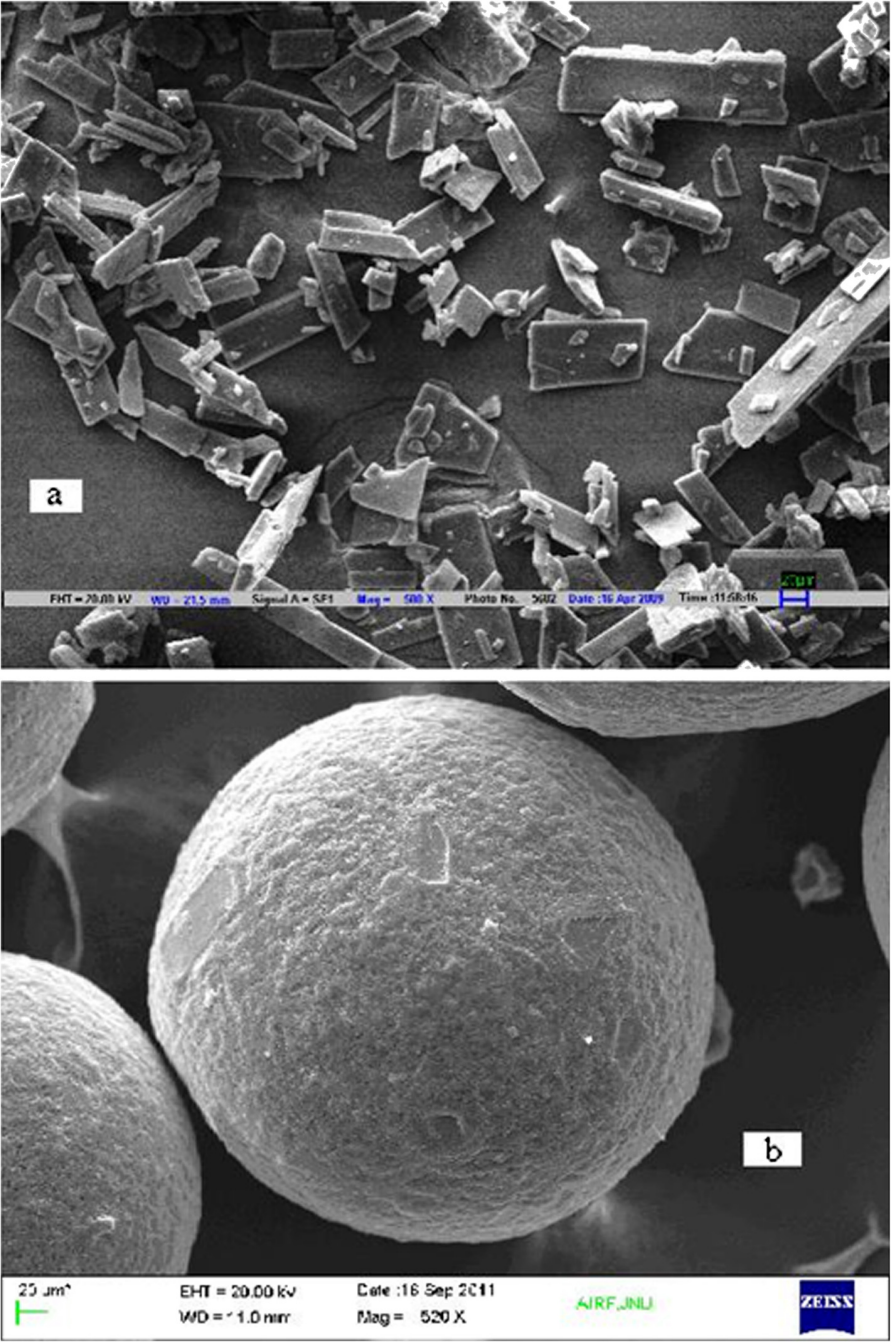
Scanning Electron Micrographs of a) Itopride HCl Crystals, b) Itopride HCl-loaded Microspheres (batch E3).

Scanning Electron Microscopy (SEM) analysis of Itopride HCl showed existence of characteristic prism like and needle like crystals as well as broad particle size distribution (Figure 2a). The structural and surface morphology of batch (E3) of microspheres having drug and polymer ratio of 1:2 showed the regularly spherical nature of microspheres with a narrow size distribution (Figure 2b) thereby confirming the encapsulation of Itopride HCl.

**Figure 3:**
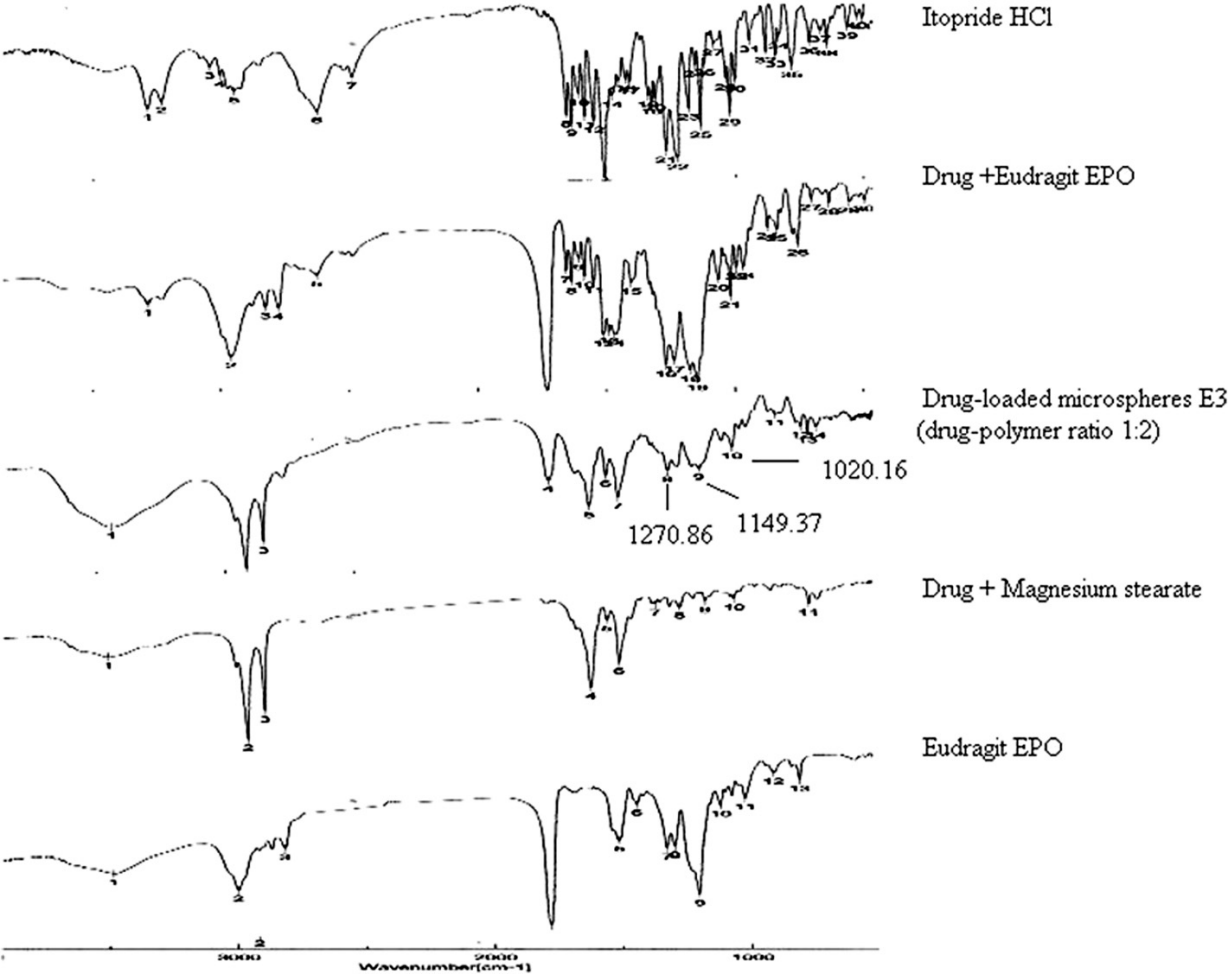
FTIR of Itopride HCl, Drug + Eudragit EPO, Drug-loaded microspheres E3 (drug-polymer ratio 1:2), Drug + Magnesium stearate, Eudragit EPO.

FTIR spectra of Itopride HCl, Eudragit EPO, physical admixture of Itopride HCl plus Eudragit EPO and drug loaded microspheres were recorded (Figure 3). FTIR spectrum of Itopride HCL showed characteristic peaks such as 1267.97 cm^-1^ for C-O-C asymmetrical ether stretching (alkyl stretching), 1028.84 cm^-1^ for C-O-C asymmetrical ether stretching (aryl ethers), 3281.29 cm^-1^ and 3226.33 cm^-1^ for NH stretching, 1631.48 cm^-1^ for NH bending, 1651.73 cm^-1^ for C = O stretching, 1147.44 cm^-1^ for C-N stretching and 2942.84 cm^-1^ and 2965.02 cm-1 for C-H. FTIR spectrum of microspheres showed some of the characteristic peaks of Itopride HCL such as 1270 cm^-1^ for C-O-C asymmetrical ether stretching (alkyl stretching), 1020 cm^-1^ for C-O-C asymmetrical ether stretching (aryl ethers), 1149 cm^-1^ for C-N stretching, thus confirming that no interaction of drug occurred with the components of the formulation. The FTIR spectrum of the physical admixture of drug plus polymer showed no significant shift or reduction in intensity of peaks of Itopride HCl at 1268.93 cm^-1^, 3281.29 cm^-1^, 1631.48 cm^-1^, 1651.73 cm^-1^, 1148 cm^-1^ and 2959.23 cm^-1^. FTIR spectroscopic studies indicated that the drug is compatible with the polymer.

**Figure 4:**
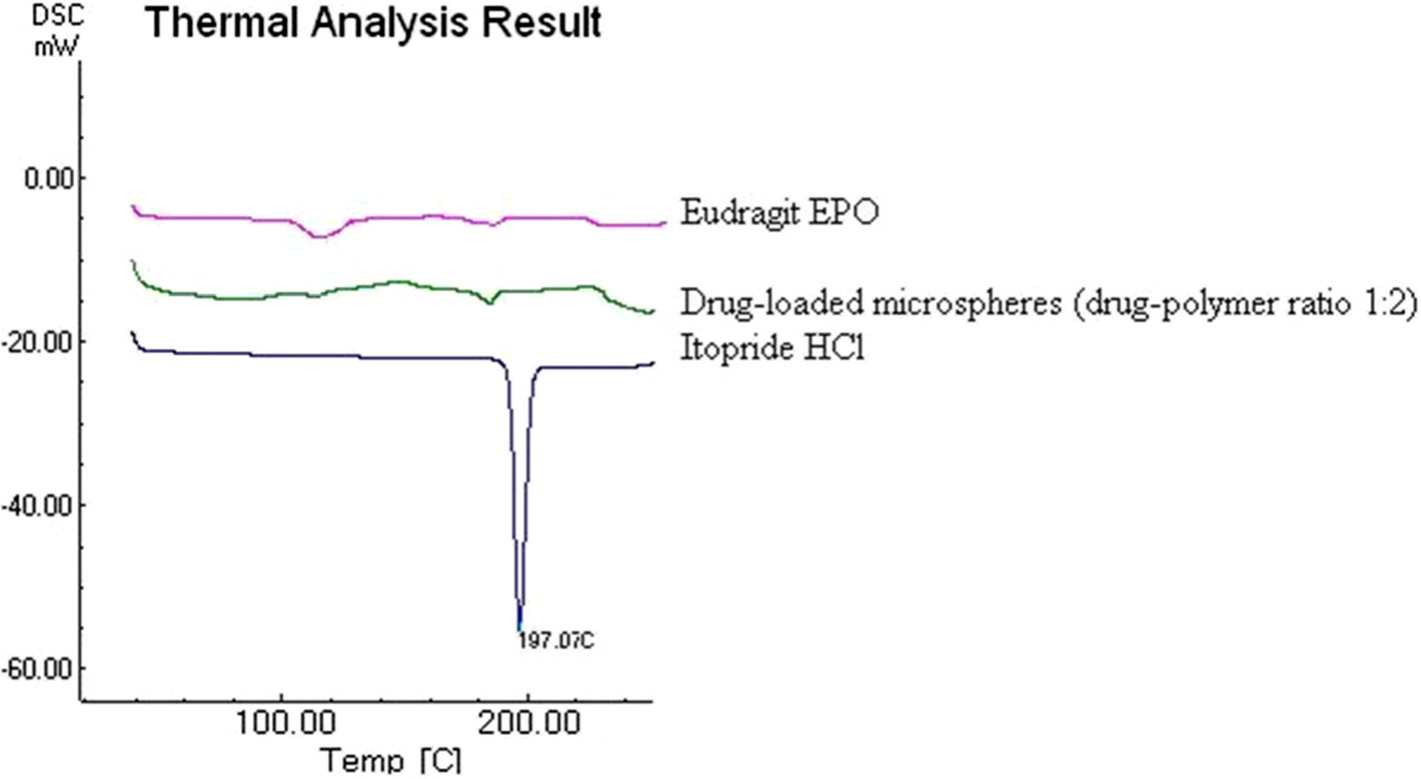
DSC of Eudragit EPO, Drug-loaded microspheres (drug-polymer ratio 1:2), Itopride HCl.

In order to check chemical interaction between drug and polymer, thermal analysis was carried out by using DSC. The melting point of drug was confirmed from the endothermic peak of Itopride at 197°C in DSC analysis. DSC thermograms of Itopride HCl, Eudragit EPO and drug-loaded microspheres (E3) showed that there were no changes in the endotherms (Figure 4). The drug exhibited a small melting endotherm in the drug-loaded microspheres (E3) formulation. These slight changes in the melting endotherm of the drug may be attributed to the mixing process, which lowers the purity of each component in the mixture, thus resulting in slightly broader and lower melting points, but not truly representing any incompatibility [31].

During formulation of orodispersible tablets of taste masked microspheres, three formulations with varying concentration of superdisintegrant: crospovidone (5, 7 and 10%w/w) were chosen on the basis of disintegration time of tablets (Table 4). Increasing the amount of Crospovidone from 5 (F1) to 7%w/w (F2) resulted in a decrease in the disintegration time of the tablets from 36 sec to 18 sec. However, further increase in the concentration to 10%w/w (F3) led to increase in disintegration time to 24 sec probably due to higher water requirement by a larger amount of Crospovidone, which consequently transformed into swelling force for rapid disintegration of the tablet. The obtained results were similar to the findings of Khan et al., 2007 [3] and Patel et al., 2004 [32]. Therefore, the suitable concentration of crospovidone was found to be 7%w/w for formulation of orodispersble tablet that showed minimal disintegration time of 18 seconds. Water insoluble diluents such as microcrystalline cellulose and dicalcium phosphate were not used in the study as they are expected to cause an unacceptable feeling of grittiness in the mouth. Among the soluble diluents, mannitol which is a hexahydric alcohol related to mannose was also used in the tablet formula as taste masking agent. Mannitol was also selected considering its advantages in terms of easy availability and negative heat of dissolution.

**Table 4:** Composition of Itopride HCl Orodispersible tablet formulations

**Tablet ingredients (mg)/Formulation code**	**F-1**	**F-2**	**F-3**
Microspheres containing Itopride HCl equivalent to 50 mg	196.6	196.6	196.6
Crospovidone (XL10)	20	28	40
Mannitol (Pearlitol)	173.4	165.4	153.4
Sodium stearyl fumarate	4	4	4
Aspartame	2	2	2
Talc	4	4	4
Total weight	400	400	400

The tablets prepared by direct compression method were found to be free from capping, chipping and sticking. The prepared tablets were evaluated for various physical parametric tests (Table 5). The thickness of all the tablets was found in range of 4.18 to 4.24 mm and was within the prescribed limits of IP 1996 (±5%) [30]. Hardness of the all tablets was between 4.5–5.7 Kg/cm^2^ during compression and was considered optimum for ODTs [32]. The average weights of tablets were found to be 399–401 mg. The acceptable weight range is ± 5% as per IP [22] for uniformity of weight thus indicating consistency in the preparation of the tablets and minimal batch to batch variation. The friability of all the formulations was found to be between 0.55 to 0.74%, which was found to be with in the pharmacopoeial requirement [33] (i.e. not more than 1%) indicating good mechanical resistance of tablet sufficient to withstand the rigors of transportation and handling. The drug content estimation data for all the batches were also found to be within the pharmacopoeial limit (i.e. 95.52 to 98.38%).

**Table 5:** Evaluation of Itopride HCl Orodispersible tablets* (Mean ± SD, n = 6)

**Evaluation parameters/Formulation code**	**F-1**	**F-2**	**F-3**
Weight Variation (mg)	399.8 ± 0.83	400 ± 2.34	401.2 ± 2.38
Hardness (Kg/cm^2^)	4.9 ± 0.5	4.5 ± 0.7	5.7 ± 0.4
Thickness (mm)	4.22 ± 0.03	4.24 ± 0.04	4.18 ± 0.02
Friability (%)	0.74 ± 0.04	0.55 ± 0.05	0.63 ± 0.01
Drug content (%)	95.52 ± 0.07	98.38 ±0.09	96.86 ± 0.02
Water Absorption ratio (%)	78.87 ± 1.14	84.72 ±1.10	74.47 ±1.81
Wetting time (sec)	32 ±2	16 ± 1	20 ± 2
In vitro Dispersion time (sec)	18 ± 1	9 ± 1	14 ± 0.5
In vitro Disintegration time (sec)	36	18	24
Moisture uptake (%)	0.71 ± 0.13	0.75 ± 0.25	0.82 ± 0.19

Wetting time is used as an indicator of the ease of the tablet disintegration in buccal cavity. It was observed that wetting time of tablets was in the range of 16 to 32 seconds which is desirable for ODT. Water absorption ratio, which is an important criteria for understanding the capacity of disintegrants to swell in presence of little amount of water, was calculated. It was found to be in the range of 74–84% which was considered to be optimum for an oro-dispersible tablet. Moisture uptake by tablets was found to be in the range of 0.71–0.82% and was considered satisfactory for an oro-dispersible tablet. All the formulations complied with the in vitro dispersion and disintegration time requirement of 60 sec for orodispersible tablets as per European Pharmacopoeia [34]. The formulation F2 containing crospovidone (7%w/w) had the least dispersion and disintegration time of 9 and 18 sec respectively.

Thus, formulation F2 possessed good disintegrating property among all formulations which was evaluated for in vitro disintegration, wetting time, in vitro dispersion time and in vivo studies.

**Figure 5:**
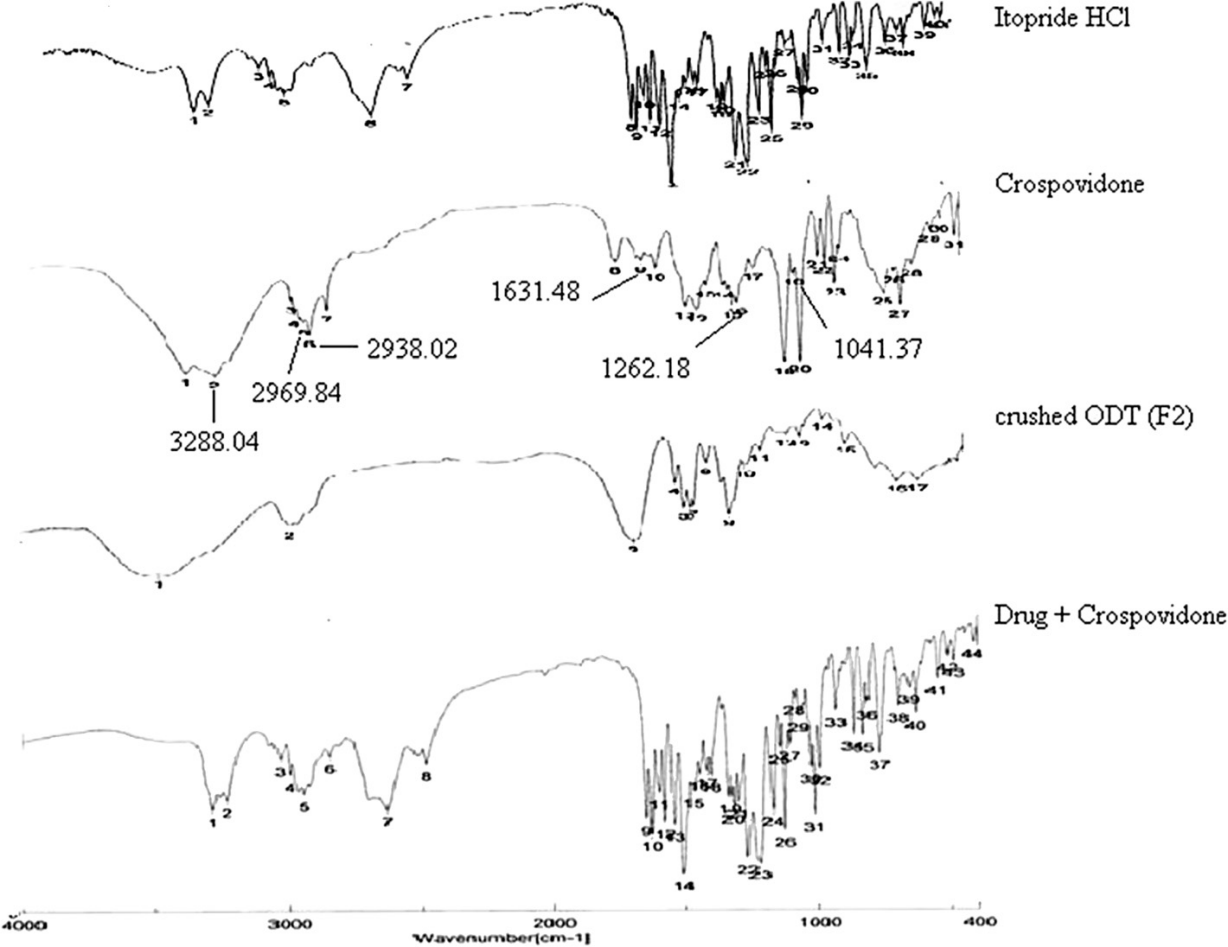
FT-IR of Itopride HCl, Crospovidone, crushed ODT (F2), Drug + Crospovidone.

Itopride HCl, crospovidone, crushed ODT (F2) and physical ad-mixture of drug plus Crospovidone was characterized by FTIR spectral analysis for any physical as well as chemical alteration of the drug characteristics (Figure 5). From the results, it was concluded that there was no interference in the functional groups as following principle peaks of the Itopride HCl such as at 3288.04 cm^–1^, 2969.84 cm^–1^, 2938.02 cm^–1^, 1631.48 cm^–1^, 1262.18 cm^–1^ and 1041.37 cm^–1^ were found in the spectra of the crushed ODT. All the peaks of Itopride HCl were found to be unaltered in the spectra of the drug-crospovidone physical admixture.

**Figure 6:**
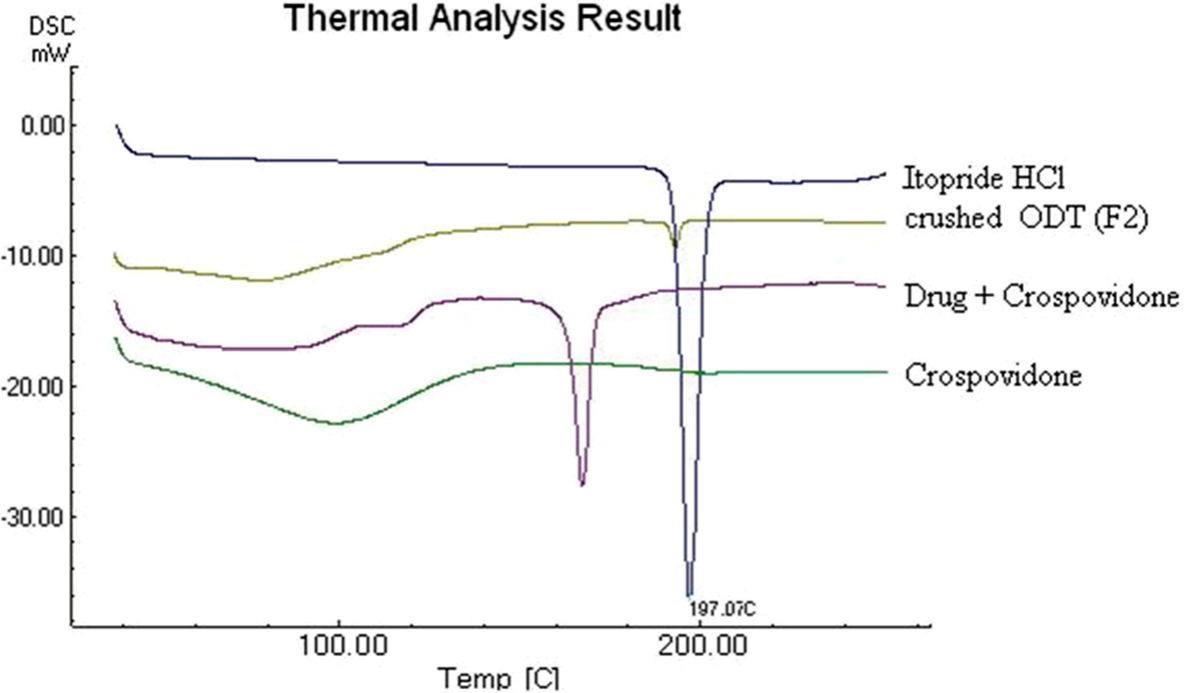
DSC of Itopride HCl, crushed ODT (F2), Drug + Crospovidone, Crospovidone.

In DSC thermal analysis, Itopride HCl exhibited melting peak at 197.07°C, while in formulation F2 the drug exhibited a small melting peak at 195.80°C. These slight changes in the melting endotherm of the drug may be attributed to the mixing process, which lowers the purity of each component in the mixture, thus resulting in slightly broader and lower melting points, but not truly representing any incompatibility [31]. However, appearance of no new peak and only a slight shift in peak of drug + crospovidone, suggested absence of interaction between drug and other excipients (Figure 6).

**Figure 7:**
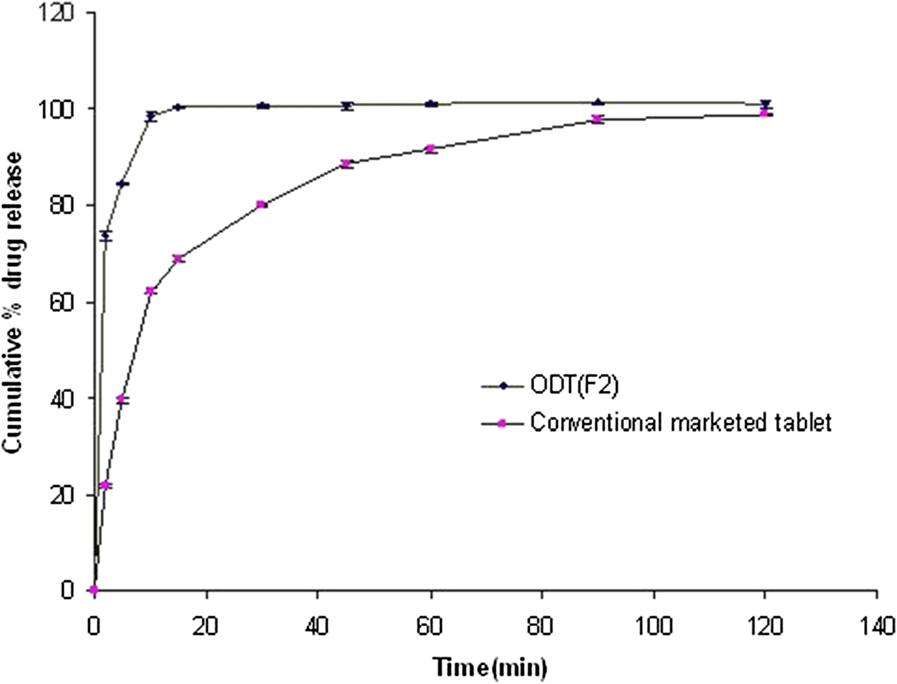
In-Vitro release profile of conventional marketed tablet (Ganaton) and formulation (F2) prepared using Crospovidone 7% and Itopride; Eudragit ratio of 1:2 in pH 1.2 HCl medium; errors bar indicate S.D, n = 3.

From the cumulative percentage drug release of ODT formulation F2 and conventional tablet formulation (GANATON) available in the market, it was observed that in the first 2 min, only 21.7% drug was released from GANATON tablet while it was 73.6% in case of ODT F2 (Table 6). At the end of 10 min, 98.5% drug was released from F2 as compared to conventional tablet formulation in which only 62.2% drug was released (Figure 7). Thus, the release rate of Itopride hydrochloride was enhanced by formulating ODTs by using crospovidone (7%w/w) as superdisintegrant. According to the FDA guidance, value of similiarity factor (*f*2) between 50 and 100 ensure sameness or equivalence of the two dissolution profiles. The vaue of similiarity factor was found to be 52 which indicate comparative equivalence with reference formulation.

**Table 6:** Dissolution profile of Orodispersible tablets (F2) and conventional tablet (Ganaton)*(Mean ± SD, n = 3)

**Time (min)**	**Cumulative percentage drug release**	
	**ODT Batch (F2)**	**Conventional tablet (Ganaton)**
0	0	0
2	73.6 ± 0.4	21.7 ± 0.9
5	84.6 ± 0.6	39.4 ± 0.2
10	98.5 ± 0.5	62.2 ± 0.9
15	100.5 ± 0.6	69.0 ± 0.2
30	100.7 ± 0.2	80.0 ± 0.4
45	100.7 ± 0.8	88.6 ± 0.9
60	101.3 ± 0.7	91.6 ± 0.4
90	101.4 ± 0.8	97.9 ± 0.3
120	101.1 ± 0.2	98.9 ± 0.8

**Figure 8:**
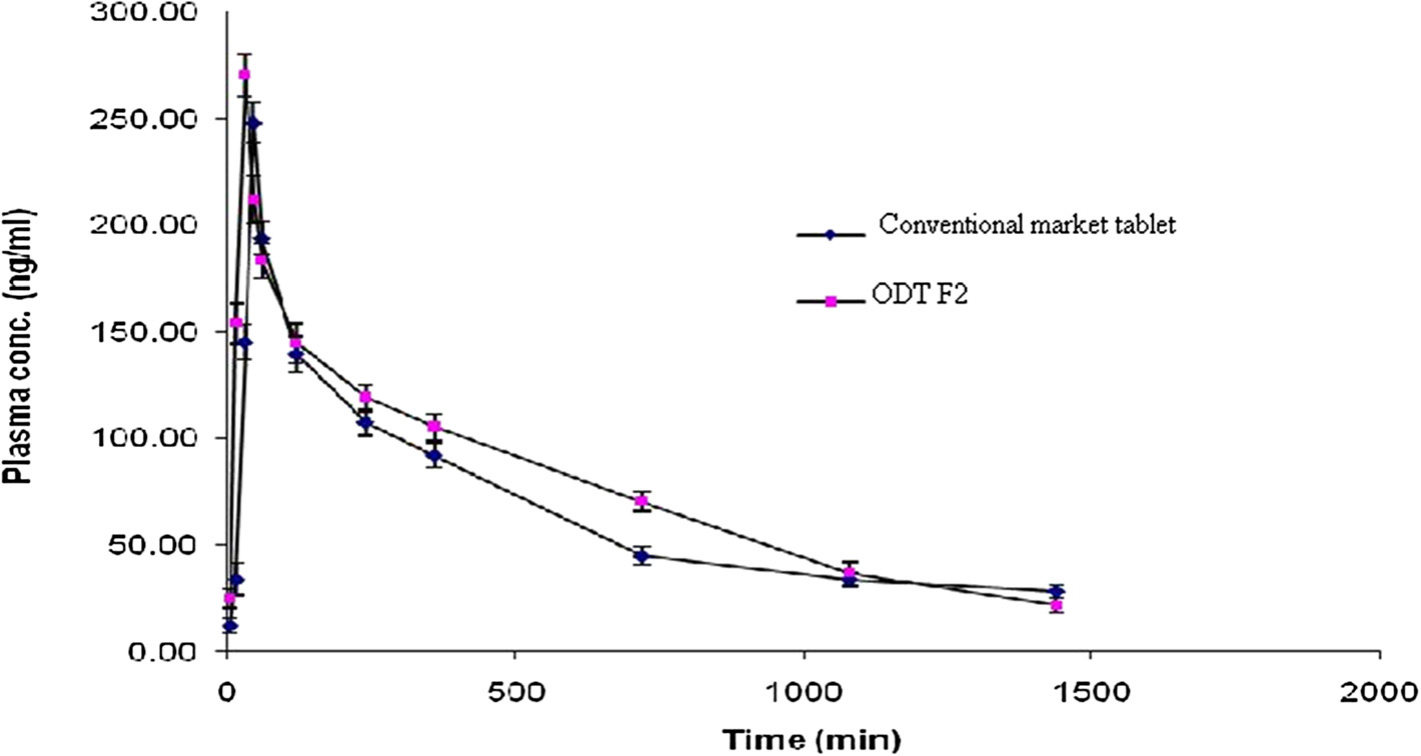
In vivo plasma profile comparison of GANATON and F2 prepared using crospovidone 7% and Itopride; Eudragit ratio of 1:2 in albino rabbits; errors bar indicate S.D, n = 3.

Further, the formulation F2 and GANATON were subjected to in vivo pharmacokinetic studies to assess the bioequivalence. The mean plasma concentration as assessed by HPTLC as a function of time after oral administration of single animal dose of Itopride HCl immediate release tablets (GANATON) and oral disintegrating tablet (F2) is shown in Figure 8. The pharmacokinetic parameters as assessed by HPTLC for both formulations are illustrated in Table 7. The absorption from oral disintegrating tablet (F2) was faster compared to GANATON both showing T_max_ of 30 and 45 min respectively. The corresponding drug plasma concentration in rabbits after administration of F2 and Marketed tablet was 270 ng/ml and 248 ng/ml respectively.

**Table 7:** Pharmacokinetic parameters of Orodispersible tablet (F2) and conventional tablet (Ganaton) in albino rabbits* (Mean ± SD, n = 3)

**Parameter**	**Formulation F2**	**Ganaton tablet**
Cmax (ng/ml)	270 ± 4.5	248 ± 5.6
Tmax (min)	30	45
AUC_0-t_ (ng.h/ml)	123.98 ± 6.3	107.78 ± 5.3

The preliminary results of stability studies carried out on formulation (F2) at 40°C/75% RH for a specific time period upto 30 days are given in Table 8. The dispersion time after 1, 2, 3 and 4 weeks of storage were found to be within USFDA limits [35]. Also, no significant loss in the drug content was found at the end of one month. Hardness and friability values were also found to be within the pharmacopoeial range after 1 month storage at 40°C.

**Table 8:** Stability studies of Orodispersible tablets ((Mean ± SD, n = 6)

**Time**	**Evaluation parameters (F2)**			
	**Hardness (kg/cm**^**2**^**)**	**Drug content %**	**Friability (%)**	**In-vitro Dispersion time (sec)**
After 1 week	4.2 ±0.7	97.65 ± 0.04	0.50 ± 0.09	9 ± 1
After 2 week	4.1 ±0.9	95.99 ±0.04	0.47 ± 0.05	8 ± 0.5
After 3 week	4.0 ±0.6	95.47 ± 0.08	0.45 ± 0.01	8 ± 1
After 4 week	3.9 ±0.5	94.80 ± 0.02	0.42 ± 0.03	7 ± 0.9

## Conclusions

This study demonstrated that the prepared Itopride HCl oral disintegrating tablets possess short in vitro disintegration time and improved dissolution patterns as well as pharmacokinetic behavior in rabbits compared to the conventional product available in market. Therefore, it can be concluded that taste masking and rapid disintegration of tablets formulated in this investigation may possibly help in administration of Itopride HCl in a more palatable form without water during emesis. Thus, the present drug delivery technology could be expected to have higher patient compliance over conventional tablets thereby necessitating the extension of this technology to development of other potential drug candidates.

## Competing interests

The authors declare that they have no competing interests.

## Authors’ contribution

SS, SM, SSA read and approved the final manuscript.
